# Comparing type 1 and type 2 error rates of different tests for heterogeneous treatment effects

**DOI:** 10.3758/s13428-024-02371-x

**Published:** 2024-03-20

**Authors:** Steffen Nestler, Marie Salditt

**Affiliations:** University of Münster, Institut für Psychologie, Fliednerstr. 21, 48149 Münster, Germany

**Keywords:** Causality, Heterogeneous treatment effects, Randomization tests, Heterogeneous regression

## Abstract

Psychologists are increasingly interested in whether treatment effects vary in randomized controlled trials. A number of tests have been proposed in the causal inference literature to test for such heterogeneity, which differ in the sample statistic they use (either using the variance terms of the experimental and control group, their empirical distribution functions, or specific quantiles), and in whether they make distributional assumptions or are based on a Fisher randomization procedure. In this manuscript, we present the results of a simulation study in which we examine the performance of the different tests while varying the amount of treatment effect heterogeneity, the type of underlying distribution, the sample size, and whether an additional covariate is considered. Altogether, our results suggest that researchers should use a randomization test to optimally control for type 1 errors. Furthermore, all tests studied are associated with low power in case of small and moderate samples even when the heterogeneity of the treatment effect is substantial. This suggests that current tests for treatment effect heterogeneity require much larger samples than those collected in current research.

In the last decades, psychologists have conducted a large number of randomized controlled trials and observational studies to test the effectiveness of specific interventions, such as, for example, an educational support program or a psychotherapy (Kravitz et al., 2004). In almost all of these studies, the parameter of interest is the average treatment effect, a measure of the overall impact of treatment. However, researchers and practitioners know that treatment effects can be highly heterogeneous across study participants. For example, students with a low social status may benefit more from an educational support program than students with a higher social status, or certain patients may respond more to a specific treatment than other patients.

Knowing the variables that are responsible for the heterogeneity in treatment effects is very interesting from an applied perspective because this would allow to better tailor the results of randomized controlled trials to particular persons (e.g., to identify the right educational support program for a student or the right treatment for a patient; see Deaton & Cartwright, 2018, but also Cook, 2018). Hence, there is a growing interest in statistical approaches that allow to detect whether – and if so, why – treatment effects vary in randomized controlled trials. Specifically, multiple methods have been suggested for detecting how the treatment effect varies based on variables that are measured prior to treatment. Among these approaches are standard linear methods such as the moderated regression model (Cox, 1984), but also different machine learning methods such as causal forests (Athey et al., 2019), causal boosting (Powers, Qian, and et al., 2018), and various meta-learners (e.g., the S-Learner, the T-Learner, or the X-Learner, see Künzel et al., 2019; Salditt et al., 2023; Wendling et al., 2018).

However, in some settings, the relevant variables may not have been measured, so that these methods cannot be applied. Then, researchers may simply want to assess whether the average treatment effect observed in their randomized controlled trial varies across participants to such a degree that it is of substantive importance. If it does not, this would indicate that the treatment can be applied to all individuals. Conversely, if it does vary, this would indicate the need for further research to investigate which variables are driving the heterogeneity. A number of tests have been proposed to assess the null hypothesis of homogeneity of treatment effects in the causal inference literature to answer this question. Despite the increasing focus on heterogeneity of treatment effects, only some of these tests are known in psychology (see Kaiser et al., 2022 for a meta-analytic application of one of these tests), and to date there are no simulation studies that have examined and compared the performance of all of these tests in different settings.

In this article we aim to present the different tests suggested in the causality literature for detecting heterogeneous treatment effects. Furthermore, we conducted a simulation study to examine their performance subject to the amount of treatment effect heterogeneity, the sample size, and the inclusion of an additional covariate when performing the test. In the following, we first introduce the average treatment effect using the potential outcomes framework (Imbens & Rubin, 2015). We then describe the different test procedures to test the null hypothesis that the average treatment effect is constant across persons. Afterwards, we present the results of the simulation study, and then conclude with a discussion of the results and questions for future research.

## Potential outcome framework and heterogeneous treatment effects

We use the potential outcome framework to define homogeneous and heterogeneous treatment effects (see Hernan & Robins, 2020; Imbens & Rubin, 2015; Rosenbaum, 2010 for introductions). To this end, let $A_i$ be the binary treatment variable with 0 indicating that person *i* is in the control group and 1 that she is in the experimental group. The potential outcome corresponding to the outcome person *i* would have experienced had she received the treatment is denoted by $Y_{i}(1)$ and the outcome had she been in the control condition is $Y_{i}(0)$. The causal effect of the treatment for the *i*th person then is

1 $$\begin{aligned} \tau _{i} = Y_{i}(1) - Y_{i}(0). \end{aligned}$$

The stable unit treatment value assumption (SUTVA, see Imbens & Rubin2015, 2015; Rosenbaum, 2002) entails that the observed outcome equals the potential outcome under the condition actually received:

2 $$\begin{aligned} Y_{i} = A_{i} \cdot Y_{i}(1) + (1 - A_{i}) \cdot Y_{i}(0). \end{aligned}$$

Since a single person *i* can only be assigned to either the experimental or the control group, only $Y_{i}(1)$ or $Y_{i}(0)$ can be observed, such that it is impossible to observe $\tau _{i}$ (see Holland, 1986). However, under certain additional assumptions, such as the assumption that the potential outcomes are independent from treatment (i.e., $A_{i} \perp \!\!\!\!\perp \lbrace Y_{i}(0),Y_{i}(1) \rbrace$), we can estimate the average of each potential outcome using the average of the observed outcome values in the experimental and control group, respectively:

3 $$\begin{aligned} \mathbb {E}[Y_{i}(0)]&= \mathbb {E}[Y_{i}|A_{i} = 0] = \mu _0 \nonumber \\ \mathbb {E}[Y_{i}(1)]&= \mathbb {E}[Y_{i}|A_{i} = 1] = \mu _1. \end{aligned}$$

This in turn allows to estimate the average of the individual treatment effects:

4 $$\begin{aligned} \mathbb {E}[\tau _{i}] = \mu _1 - \mu _0. \end{aligned}$$

When the treatment effect is homogeneous across all persons, that is, $\tau _{i} = \tau$ for all $i = 1, \dots , n$, the average treatment effect equals the constant effect, $\mathbb {E}[\tau _{i}] = \tau$. Thus, the treatment increases the *mean* difference between the experimental and control group by amount $\tau$:

5 $$\begin{aligned} \mathbb {E}[Y_{i}(1)] = \mathbb {E}[Y_{i}(0)] + \tau . \end{aligned}$$

## Tests for heterogeneous treatment effects

The goal of all test procedures suggested in the causal inference literature is to test the null hypothesis that the treatment effect is constant for all persons:

6 $$\begin{aligned} H_{0}: Y_{i}(1) = Y_{i}(0) + \tau . \end{aligned}$$

The proposed tests differ in the sample statistics they use and in whether they make distributional assumptions for the potential outcomes. Concerning sample statistics, the different tests use estimates of the variance parameters in the control and the experimental group, the empirical distribution functions of the two groups, or they compare a grid of quantiles estimated in the two groups. Concerning the distributional assumptions, some tests presume that the potential outcomes, and therefore the observed outcomes, are normally distributed, while other tests belong to the class of Fisher randomization tests (FRT) that do not rely on any distributional assumptions (Imbens & Rubin, 2015; Rosenbaum, 2002).

### Comparing variance terms

There are several ways to implement a comparison of variance terms for testing treatment effect heterogeneity. All of these tests are based on the observation that when the treatment effect is constant across persons, it does not influence the variance of the potential outcomes:

7 $$\begin{aligned} \mathbb {V}[Y_{i}(1)] = \mathbb {V}[Y_{i}(0) + \tau ] = \mathbb {V}[Y_{i}(0)] \end{aligned}$$

where the first equality follows from Eq. 1 and the second uses the fact that the variance of a constant is zero. Under the assumptions introduced above, $\mathbb {V}[Y_{i}(1)]$ and $\mathbb {V}[Y_{i}(0)]$ equal the variance of the observed values in the experimental and the control group, respectively:

8 $$\begin{aligned} \mathbb {V}[Y_{i}(1)]&= \mathbb {V}[Y_{i}|A_{i} = 1] = \sigma ^{2}_{1} \nonumber \\ \mathbb {V}[Y_{i}(0)]&= \mathbb {V}[Y_{i}|A_{i} = 0] = \sigma ^{2}_{0}. \end{aligned}$$

#### Variance ratio

Using Eq. 8, a potential test statistic to test for treatment effect heterogeneity consists of computing the ratio of the two estimates of the aforementioned variance terms:

9 $$\begin{aligned} T_{V} = \frac{\hat{\sigma }^{2}_{1}}{\hat{\sigma }^{2}_{0}}. \end{aligned}$$

When we assume that each potential outcome is normally distributed, $T_{V}$ is *F*-distributed with degrees of freedom $n_{1} - 1$ and $n_{0} - 1$ and testing $T_{V}$ equals the *F*-test for two variance terms (e.g., Casella & Berger, 2002). Alternatively, one can use a heterogeneous regression model in which the residual variance is modeled as a function of the treatment variable (Bloome & Schrage, 2021; Western & Bloome, 2009):

10 $$\begin{aligned} y_{i}&\sim \mathcal {N}(\beta _0 + \beta _1 \cdot A_i, \sigma ^{2}_{\epsilon _i} ) \nonumber \\ \text {log}(\sigma ^{2}_{\epsilon _i} )&= \gamma _0 + \gamma _1 \cdot A_i \end{aligned}$$

Here, the estimate $\hat{\gamma }_1$ measures the difference between the variance of the two groups on a log-scale and a test statistic $T_{\gamma }$ is obtained by dividing $\hat{\gamma }_1$ by its standard error. The resulting test statistic can be used to test the presence of a heterogeneous treatment effect (Bloome & Schrage, 2021), and we will simply refer to this test as $T_{\gamma }$ in the simulation study reported later. Importantly, $\hat{\gamma }_1$ equals $T_{V}$ after exponentiation, which is why we only consider $T_{\gamma }$ and not the *F*-test for $T_{V}$ as variance-ratio-based test that assumes normally distributed data, because the two tests yield the same decision concerning the null hypothesis.

A test that does not rely on such distributional assumptions can be obtained when using $T_{V}$ in a Fisher randomization test (FRT, Imbens & Rubin, 2015; Rosenbaum, 2002; 2010). The basis of the FRT is that – given the aforementioned null hypothesis – the missing potential outcome values can be imputed assuming that we know the treatment effect. Thus, when person *i* belongs to the experimental group, her observed value is $Y_i = Y_i(1)$ and the missing potential outcome is $Y_i(0) = Y_i(1) - \tau = Y_i - \tau$. Alternatively, when person *i* is in the control group, her missing potential outcome is $Y_i(1) = Y_i + \tau$.

Using this, the FRT constructs a sampling distribution of the test statistic ($T_{V}$ in this case) by randomly permuting the observed values of the *treatment variable*. To illustrate, when the observed treatment values are 0, 1, 1, 0, 0, and 1 for person one to six, a randomly permuted treatment variable would be 1, 0, 0, 1, 1, and 0. The test statistic is then computed again using the group assignments in the permuted treatment variable. This process is repeated a large number of times (e.g., *B* = 1000) and the resulting distribution of the test statistic (e.g., the distribution of the $T_{V}$ values) is used to obtain a *p* value by computing the relative frequency of the values of the test statistic that are greater than the observed test statistic:

11 $$\begin{aligned} p = \frac{1}{B} \sum _{j = 1}^{B} I(T_{j} > T_{\textrm{obs}}) \end{aligned}$$

where $T_{\textrm{obs}}$ is the observed test statistic (i.e., $T_{V}$ here) and $T_j$ is the value of the test statistic in the *j*th randomization sample. As said above, the basis of the FRT is that given the null hypothesis of a constant treatment effect, the missing potential outcome values can be imputed assuming that one knows the true average treatment effect. In case of $T_{V}$ the assumption of knowing the true treatment effect is not important, because the test compares two variance parameters that – given the null hypothesis – are not affected by the ‘constant’ $\tau$.

#### Variance difference

$T_{V}$ and $T_{\gamma }$ use the ratio of the two variance terms. Another way to test the null hypothesis of a constant treatment effect consists of taking the absolute difference between the two variance terms

12 $$\begin{aligned} T_{D} = |\hat{\sigma }^{2}_{1} - \hat{\sigma }^{2}_{0}| \end{aligned}$$

and to use $T_{D}$ in a FRT (we refer to this test as $T_{D}$ in the simulation study reported later). Alternatively, when one assumes normally distributed potential outcomes, one can implement a test of the variance difference in a random coefficient regression model or in the multiple group structural equation model framework. In a random coefficient regression model, the slope of a predictor variable is allowed to vary across individuals (as in the case of a multilevel model), although only one score is available for a single participant (in econometrics this model is called the Hildreth–Houck model, see Hildreth & Houck, 1968; Muthén et al., 2017). Applied to the present context, the slope of the treatment variable is modeled to differ between persons:

13 $$\begin{aligned} y_i&= \beta _0 + \beta _{1i} \cdot A_i + \epsilon _{i} \nonumber \\ \beta _{1i}&= \beta _1 + u_{i} \end{aligned}$$

where $u_i$ is a normally distributed error term with expectation zero and variance $\sigma _{u}^{2}$. When the (normally distributed) error term is uncorrelated with $u_i$, the conditional variance of $y_i$ is given by

14 $$\begin{aligned} \mathbb {V}[y_{i}|A_i] = A_i \cdot \sigma _{u}^{2} + \sigma _{\epsilon }^{2} \end{aligned}$$

Thus, the variance in the control group is $\mathbb {V}[y_{i}|A_i = 0] = \sigma _{\epsilon }^{2}$ and in the experimental group the variance is $\mathbb {V}[y_{i}|A_i = 1] = \sigma _{u}^{2} + \sigma _{\epsilon }^{2}$, showing that the *difference* between the two is $\sigma _{u}^{2}$. When we fit the model to the data (using Mplus, see below and Muthen & Muthen, 1998-2017), a Wald test can then be employed to check whether the sample estimate $\hat{\sigma }_{u}^{2}$ is significantly different from zero. In the simulation study, we call this test $T_{\beta }$.

The second way to implement the variance difference test when assuming normally distributed data is to use a multiple group structural equation model (Tucker-Drob, 2011). Specifically, one fits a structural equation model to the data of the experimental group and another one to the control group and compares the fit of this unconstrained multiple group model to the fit of a constrained multiple group model in which the variance terms of the outcome variable are restricted to be equal. Formally, the fit of the two models is compared using a chi-squared difference test that is a likelihood-ratio (LR) test. The LR-test is asymptotically equivalent to a Wald-test that compares the difference between the two variance terms (Bollen, 1989; Muthén et al., 2017). Later, we abbreviate this test with $T_{LR}$.

#### Variance ratio and variance differences with rank data

All tests described so far use the observed data to compute the respective test statistic. However, in the case of testing the null hypothesis of no *average* treatment effect, simulation studies showed that a FRT that uses rank data has good error rates across different simulation conditions (Imbens & Rubin, 2015; Rosenbaum, 2002, 2010). To conduct such a rank-based FRT, one first transforms the outcome to ranks and then computes the test statistic on the resulting rank data. To examine whether the good performance of the rank-based FRTs for testing the average treatment effects against zero generalizes to the context of heterogeneous treatment effects, we also consider rank-based versions of the FRTs in the simulation study (referred to as $T^{R}_{V}$ and $T^{R}_{D}$, respectively).

### Comparing the cumulative distribution functions

A second group of tests is based on the two-sample Kolmogorov–Smirnov test statistic. The idea is to compare the marginal distribution function in the control group with the marginal distribution function in the experimental group, because under the null hypothesis of a constant treatment effect, the two distribution functions differ only by the average treatment effect $\tau$. Thus, if $\tau$ would be known, one could use (Ding et al., 2016)

15 $$\begin{aligned} T_{\textrm{KS}} = \underset{y}{\max }|\hat{F}_{0}(y) - \hat{F}_{1}(y + \tau )|, \end{aligned}$$

where $\hat{F}_{0}$ and $\hat{F}_{1}$ denotes the empirical cumulative distribution functions of the outcome in the control and experimental group, respectively. $T_{\textrm{KS}}$ presumes that the true treatment effect $\tau$ is known. Because this is not the case, an obvious fix would be to use the estimated average treatment effect $\hat{\tau }$, resulting in the ’shifted’ test statistic:

16 $$\begin{aligned} T_{\textrm{SKS}} = \underset{y}{\max }|\hat{F}_{0}(y) - \hat{F}_{1}(y + \hat{\tau })|. \end{aligned}$$

However, (Ding et al., 2016) show that the sampling distribution of $T_{\textrm{SKS}}$ is not equivalent to the sampling distribution of the standard KS test statistic (see Wasserman, 2004 for the function) and therefore yields either inflated or deflated false-positive and false-negative error rates. To deal with this problem, they suggest to use $T_{\textrm{SKS}}$ in a FRT. To this end, one first computes the potential outcomes $Y_{i}(0)$ and $Y_{i}(1)$ for each participant by *plugging in* the estimated average treatment effect $\hat{\tau }$. That is, $Y_{i}(0) = Y_i$ and $Y_{i}(1) = Y_i + \hat{\tau }$ for the persons in the control group, and $Y_{i}(0) = Y_i - \hat{\tau }$ and $Y_{i}(1) = Y_i$ for the persons in the experimental group. These potential outcomes are then used as the basis for the FRT in which $T_{\textrm{SKS}}$ is computed in each randomization sample. The resulting distribution of the $T_{\textrm{SKS}}$ scores is then used to test the null hypothesis (see Eq. 11).

Ding et al. (2016) call this the FRT-Plug in test (henceforth, FRT-PI) and argue that the procedure should yield valid results when the estimated average treatment effect is close to the true average treatment effect (e.g., when the sample size is large). However, as there are no guarantees that this is the case, Ding et al. (2016) suggest a second FRT in which not only one value for the hypothesized constant treatment effect is plugged in, but rather a range of plausible average treatment effects. Specifically, they suggest to construct a 99.9% confidence interval for the estimated average treatment effect and to use a grid of values covering this interval in the FRT. That is, for *each* of these treatment effects, the FRT is performed and one then finds the maximum *p* value over all the resulting randomization tests. When this *p* value is smaller than the significance level, one rejects the null hypothesis. Following Ding et al. (2016), we henceforth call this test FRT-CI.

### Comparing quantiles

A third test was suggested by Chernozhukov & Fernandez-Val (2005) and is based on a comparison of quantiles instead of the cumulative distribution functions. Specifically, the test is based on the observation that

17 $$\begin{aligned} F^{-1}_{1}(q) = F^{-1}_{0}(q) + \tau (q) \end{aligned}$$

where $F^{-1}_{0}$ and $F^{-1}_{1}$ is the inverse of the cumulative distribution function in the control and experimental group, respectively, *q* is a certain quantile, and $\tau (q)$ is the average treatment effect at the *q*th quantile. When the null hypothesis of a constant treatment effect is true, $\tau (q)$ would be constant across all quantiles *q*. Therefore, Chernozhukov & Fernandez-Val (2005) suggest testing the null hypothesis with a type of KS-statistic in which one first obtains estimates of the treatment effect at certain quantiles and then takes the largest difference between these values and the average treatment effect:

18 $$\begin{aligned} T_{\textrm{sub}}= \underset{q}{\max }|\hat{\tau }(q) - \hat{\tau }| \end{aligned}$$

In their implementation of the test, Chernozhukov & Fernandez-Val (2005) use quantile regression (Hao & Naiman, 2007), in which the outcome *Y* is regressed on the treatment indicator *A* at a quantile *q*, to obtain an estimate of $\hat{\tau }(q)$.

Similar to the shifted KS statistic (see Eq. 16), $T_{\textrm{sub}}$ uses an estimate of the true average treatment effect. Therefore, Chernozhukov & Fernandez-Val (2005) suggest to use a subsampling approach to obtain a valid test statistic. In subsampling, the sampling distribution of a statistic is obtained by drawing subsamples of a certain size *b* *without* replacement from the current dataset. In each subsample, the respective statistic is computed ($T_{\textrm{sub}}$ in our case) and the resulting sampling distribution is then used – similar to the bootstrap – for inferences concerning the statistic. Chernozhukov & Fernandez-Val (2005) show that their subsampling approach is asymptotically correct. They also found their approach to yield valid type 1 error rates in a simulation study.

### Comparing coefficients of variation

Finally, it was also suggested to compare the coefficients of variation (CV) instead of the variance terms to detect heterogeneous treatment effects (Nakagawa et al., 2015; Volkmann et al., 2020), because the magnitude of the variance depends on the mean (e.g., the larger the mean on a bounded scale, the lower a variable’s reachable variance). The coefficient of variation is the ratio of the standard deviation $\sigma$ of a variable to its mean $\mu$

19 $$\begin{aligned} CV = \frac{\sigma }{\mu } \end{aligned}$$

and a potential test statistic then is to compare the estimated CVs from the two groups

20 $$\begin{aligned} T_{CV} = \frac{\hat{CV}_{1}}{\hat{CV}_{0}} = \frac{\hat{\sigma }_{1}/\hat{\mu }_{1}}{\hat{\sigma }_{0}//\hat{\mu }_{0}} = \frac{\hat{\sigma }_{1}}{\hat{\sigma }_{0}} \cdot \frac{\hat{\mu }_{0}}{\hat{\mu }_{1}} \end{aligned}$$

However, a problem with using $T_{CV}$ is that the test statistic is not only affected by a heterogeneous treatment effect (e.g., $\hat{\sigma }_{1} \ne \hat{\sigma }_{0}$), but also by whether there is a nonzero average treatment effect. For instance, when $\hat{\sigma }_{1}$ = $\hat{\sigma }_{0}$ = 1 and $\hat{\mu }_{1} = \hat{\mu }_{0} + 1$, then $T_{CV}$ would be equal to 0.5. Thus, $T_{CV}$ cannot be used to test the null hypothesis of a constant treatment effect, at least when the average treatment effect is nonzero.^1^ Therefore, we do not consider this test statistic in our simulation.

### Considering covariates

So far we assumed that data is available for the treatment variable and the outcome only. However, when a randomized controlled trial is conducted in practice, researchers may also have assessed a number of person-level pretreatment covariates. When testing the average treatment effect against zero, it is well known (e.g., Murnane & Willett, 2011) that considering these covariates in the model can increase the precision of the treatment effect estimate and power. In a similar way, considering covariates may help to increase the power of the treatment heterogeneity tests.

In case of the test based on the heterogeneous regression model ($T_{\gamma }$, see Eq. 10), the random coefficient model ($T_{\beta }$, see Eq. 13), and the structural equation model ($T_{LR}$), one can consider such covariates by including them as predictors of the outcome variable.^2^ For the other tests, the outcome variable is first regressed on the covariates. The residuals of this regression can then be used in the FRT approaches or in the tests based on the cumulative distribution function. In the latter case, one then computes a ‘regression-adjusted KS statistic’ (cf., Ding et al., 2016). For the quantile-based test (i.e., $T_{\textrm{sub}}$), Koenker and Xiao (2002) suggested to estimate $\tau (q)$ in a quantile regression of the outcome on the treatment variable and the covariates and to use these estimates when computing $T_{\textrm{sub}}$.

**Table 1:** Summary of heterogeneous treatment effect tests considered in the text

Sample statistic	Distribution	Name	Brief description
Variance ratio	Normal	$T_{\gamma }$	Regression model with treatment variable as a predictor of the residual variance
	FRT - Raw data	$T_{V}$	FRT using the ratio of the variance terms estimated with the raw data
	FRT - Ranks	$T^{R}_{V}$	FRT using the ratio of the variance terms estimated with the rank data
Variance difference	Normal	$T_{LR}$	Test that compares the fit of a constrained and an unconstrained multiple group SEM
		$T_{\beta }$	Random coefficient regression model with varying slope of the treatment variable
	FRT - Raw data	$T_{D}$	FRT using the difference of the variance terms estimated with the raw data
	FRT - Ranks	$T^{R}_{D}$	FRT using the difference of the variance terms estimated with the rank data
Distribution function	FRT - Plug in	FRT-PI	FRT using the two-sample KS statistic for potential outcomes computed with the ATE
	FRT - repeated Plug in	FRT-CI	Same as FRT-PI but computed for a range of plausible ATEs
Quantiles	Subsampling	$T_{sub}$	Subsampling approach using the largest difference between estimates of the ATE at several quantiles and the overall ATE

Note. FRT = Fisher randomization test, KS = Kolmogorov–Smirnov, ATE = Average treatment effect

## The present research

The foregoing discussion showed that a number of tests were proposed in the causal inference literature to test for heterogeneity in treatment effects (see Table 1 for a summary) and simulation studies conducted so far found that *some* of the proposed tests keep their nominal type 1 error rates while also preserving low type 2 error rates. Bloome and Schrage (2021), for example, examined the performance of $T_{\gamma }$ in case of normally distributed potential outcomes and a sample size of 200 or 1000 persons per group. They found that $T_{\gamma }$ has good type 1 error rates at both sample sizes and that the type 2 error rate gets smaller the larger the size of the two groups. Ding et al. (2016) compared their proposed FRT approaches (i.e., FRT-PI and FRT-CI) with the subsampling approach of (Chernozhukov & Fernandez-Val, 2005) at sample sizes of 100 or 1000 persons in each group. When the treatment effect is homogeneous, all three tests yielded good type 1 error rates. Furthermore, the power of the tests to detect heterogeneous treatment effects was low for all three methods when the size of the samples was small. However, as expected, the power increased with increasing sample sizes and with increasing treatment effect heterogeneity.

The simulation study reported here is aimed at replicating and extending these results. Specifically, we conducted the simulation study with four purposes. First, in our review we described several tests whose suitability for testing treatment effect heterogeneity has not yet been investigated (e.g., the random coefficient model, rank-based FRTs), neither alone nor in comparison to the other tests. We wanted to compare all of the proposed tests in one simulation study, rather than evaluating a subset of the tests only. We believe that this is important because so far psychologists often compare – if at all – the variance terms to test the presence of heterogeneous treatment effects and seldomly use (rank-based) FRT versions. Thus, if the other tests have higher power, applied researchers would currently make a type 2 error unnecessarily often (i.e., they would underestimate the frequency of heterogeneous treatment effects). Second, we wanted to examine the error rates at smaller sample sizes. So far, sample sizes of 100, 200, or 1000 persons per group have been investigated. However, randomized controlled trials sometimes involve fewer persons per condition and it is thus important to evaluate the performance of the tests at such smaller sample sizes. Third, to the best of our knowledge, there is no simulation study that has investigated whether and to what extent the inclusion of covariates affects the power of the various tests. Finally, we also wanted to investigate whether (and how) the error rates are affected when variables are not normally distributed, but stem from distributions in which more extreme values can occur, or which are skewed.

## Simulation study

We performed a simulation study to assess the type 1 and type 2 error rates in different sample size conditions and in different conditions of treatment effect heterogeneity. We also varied the size of the average treatment effect and the distribution of the potential outcomes, and we examined whether the consideration of a covariate increase the power of the tests. The R code for the simulation study can be found at https://osf.io/nbrg2/.

### Simulation model and conditions

The population model that we used was inspired by the model used in Chernozhukov and Fernandez (2005) and Ding et al. (2016). Specifically, the model used to generate the data was an additive treatment effect model:

21 $$\begin{aligned} Y_{i}(0)&= \epsilon _{i} \nonumber \\ Y_{i}(1)&= Y_{i}(0) + \tau _{i} \nonumber \\ \tau _{i}&= \tau + \sigma _{\tau } Y_{i}(0). \end{aligned}$$

To manipulate the size of the heterogeneous treatment effect, we followed (Ding et al., 2016) by setting $\sigma _{\tau }$ to 0, 0.25, or 0.5, corresponding to a constant treatment effect across participants, a moderate, or a large heterogeneous treatment effect. Although no conventions with regard to the size of heterogeneous treatment effect exist, we call the two conditions moderate and large, because setting $\sigma _{\tau }$ to 0.25 (0.50) implies that the variance in the experimental group is about 1.5 (2) times larger than the variance in the control group (i.e., $\sigma ^2_{0} = 1$ vs. $\sigma ^2_{1} = 1.56$ and $\sigma ^2_{1} = 2.25$, respectively). To manipulate the type of underlying distribution, $\epsilon _{i}$ was generated from a standard normal distribution (i.e., $\epsilon _{i} \sim \mathcal {N}(0,1)$), from a *t*-distribution with 5 degrees of freedom, or from a log-normal distribution with mean 0 and standard deviation 1 (both on a log-scale). The *t*-distribution is a symmetric distribution that has thicker tails compared to the normal distribution. The chance that extreme values occur in a random sample are thus larger. The log-normal distribution, by contrast, is an asymmetric distribution. Using this distribution thus allows us to examine how skewness of the data influences the five tests. Furthermore, the average treatment effect $\tau$ was set to either 0 or 1. The latter manipulation was examined to examine whether the performance of the tests is affected by the presence of an nonzero average treatment effect and we set $\tau$ to this rather large value (i.e., in the normal case the implied *d* is 1) to not miss any effects that an (incorrect) estimation of the average treatment effect might have.

The number of participants was set to 25, 50, 100, or 250 in each group. A sample size of 25 per group is a rather small sample size for a randomized controlled trial and 250 participants per group can be considered a large sample (reflecting, for example, the growing number of multi-center studies). The other two sample sizes are in between and were included to examine the error rates in case of more moderate sample sizes. Finally, to examine the effect of including a covariate on the power of the tests, we generated a standard normal variable $X_i$ and used

22 $$\begin{aligned} Y_{i}(0) = 0.30 \cdot X_{i} + \epsilon _{i} \end{aligned}$$

to generate $Y_{i}(0)$ in the first step of the respective conditions. In the case where $\epsilon _{i}$ is drawn from a standard normal distribution, this implies that the correlation between $X_i$ and each of the potential outcomes is 0.30, which corresponds to a moderate effect.

### Tests and dependent variables

For each of the 3 (size of heterogeneity) $\times$ 3 (type of distribution) $\times$ 2 (zero versus nonzero average treatment effect) $\times$ 4 (no. of participants in a group) $\times$ 2 (zero versus nonzero covariate) = 144 conditions, we generated 1000 simulated data sets. For each condition, the 1000 replications were analyzed by computing the ten test statistics, that is, $T_{\gamma }$, $T_{V}$, $T^{R}_{V}$, $T_{LR}$, $T_{\beta }$, $T_{D}$, $T^{R}_{D}$, FRT-PI, FRT-CI, and $T_{\textrm{sub}}$.^3^$T_{V}$, $T^{R}_{V}$, $T_{D}$, and $T^{R}_{D}$ were each implemented as a FRT. We used *B* = 2000 randomization samples to obtain the sampling distribution of the statistic in each replication. This distribution was then used to obtain the *p* value for this replication. When the *p* value was smaller than $\alpha = .05$, the null hypothesis of a constant treatment effect was rejected, otherwise it was accepted.

**Table 2:** Rejection rates for different heterogeneous treatment effect tests depending on the distribution of the potential outcomes, amount of treatment effect variation ($\sigma _{\tau }$), and sample size when the average treatment effect is zero and no covariates are considered

		$\sigma _{\tau }$ = 0				$\sigma _{\tau }$ = 0.25				$\sigma _{\tau }$ = 0.50			
Distribution	Test	25	50	125	250	25	50	125	250	25	50	125	250
*N*(0,1)	$T_{\gamma }$	0.055	0.051	0.052	0.056	0.198	0.358	0.615	0.942	0.515	0.808	0.983	1.000
	$T_{V}$	0.043	0.043	0.051	0.057	0.165	0.332	0.578	0.942	0.434	0.777	0.980	1.000
	$T_{V}^{R}$	0.044	0.046	0.054	0.059	0.148	0.265	0.471	0.864	0.345	0.664	0.941	1.000
	$T_{LR}$	0.056	0.052	0.052	0.057	0.201	0.361	0.618	0.942	0.518	0.810	0.984	1.000
	$T_{\beta }$	0.033	0.026	0.027	0.032	0.189	0.342	0.591	0.944	0.473	0.777	0.980	1.000
	$T_{D}$	0.045	0.042	0.051	0.057	0.170	0.329	0.578	0.942	0.431	0.780	0.979	1.000
	$T_{D}^{R}$	0.044	0.044	0.053	0.059	0.143	0.266	0.469	0.864	0.343	0.667	0.941	1.000
	FRT-PI	0.042	0.042	0.037	0.044	0.097	0.129	0.248	0.513	0.160	0.341	0.779	0.977
	FRT-CI	0.006	0.017	0.022	0.034	0.014	0.071	0.194	0.463	0.040	0.247	0.685	0.968
	$T_{\textrm{sub}}$	0.006	0.012	0.014	0.037	0.029	0.098	0.164	0.640	0.102	0.352	0.633	0.996
*t*(5)	$T_{\gamma }$	0.145	0.211	0.214	0.236	0.281	0.395	0.561	0.825	0.485	0.724	0.907	0.991
	$T_{V}$	0.044	0.053	0.051	0.055	0.122	0.186	0.328	0.627	0.300	0.524	0.757	0.960
	$T_{V}^{R}$	0.048	0.063	0.044	0.053	0.116	0.209	0.380	0.761	0.296	0.562	0.858	0.997
	$T_{LR}$	0.149	0.213	0.216	0.236	0.286	0.403	0.562	0.827	0.489	0.726	0.907	0.991
	$T_{\beta }$	0.018	0.021	0.027	0.032	0.119	0.173	0.302	0.605	0.285	0.485	0.715	0.941
	$T_{D}$	0.048	0.052	0.051	0.054	0.126	0.186	0.327	0.626	0.296	0.527	0.757	0.960
	$T_{D}^{R}$	0.047	0.061	0.042	0.053	0.113	0.209	0.380	0.761	0.290	0.562	0.859	0.997
	FRT-PI	0.032	0.052	0.052	0.048	0.070	0.084	0.169	0.341	0.121	0.243	0.515	0.879
	FRT-CI	0.007	0.016	0.028	0.035	0.011	0.036	0.108	0.264	0.022	0.128	0.417	0.828
	$T_{\textrm{sub}}$	0.009	0.013	0.005	0.011	0.018	0.047	0.047	0.259	0.069	0.172	0.284	0.820
log-*N*	$T_{\gamma }$	0.517	0.574	0.625	0.655	0.545	0.631	0.665	0.768	0.587	0.690	0.756	0.892
	$T_{V}$	0.052	0.061	0.056	0.063	0.061	0.083	0.112	0.176	0.137	0.202	0.274	0.442
	$T_{V}^{R}$	0.057	0.058	0.049	0.059	0.375	0.664	0.937	1.000	0.736	0.965	0.999	1.000
	$T_{LR}$	0.519	0.575	0.626	0.655	0.548	0.632	0.666	0.768	0.591	0.693	0.757	0.892
	$T_{\beta }$	0.030	0.019	0.023	0.017	0.049	0.052	0.064	0.119	0.090	0.111	0.168	0.315
	$T_{D}$	0.052	0.056	0.056	0.063	0.091	0.090	0.117	0.179	0.183	0.222	0.289	0.447
	$T_{D}^{R}$	0.044	0.044	0.053	0.059	0.143	0.266	0.469	0.864	0.343	0.667	0.941	1.000
	FRT-PI	0.158	0.136	0.096	0.096	0.226	0.254	0.302	0.470	0.342	0.444	0.630	0.854
	FRT-CI	0.022	0.032	0.050	0.028	0.024	0.094	0.180	0.376	0.052	0.160	0.478	0.778
	$T_{\textrm{sub}}$	0.015	0.009	0.007	0.009	0.015	0.024	0.011	0.071	0.021	0.052	0.100	0.408

Note. *N*(0,1) = standard normal distribution, *t*(5) = *t*-distribution with 5 degrees of freedom, log-*N* = log-normal distribution. $T_{\gamma }$: variance ratio test with heterogeneous regression model, $T_{V}$: FRT with raw data based variance ratios, $T_{V}^{R}$: FRT with rank data based variance ratios, $T_{LR}$: variance difference test with LR-Test from multiple group SEM, $T_{\beta }$: variance difference test with random coefficient regression model, $T_{D}$: FRT with raw data based variance differences, $T_{D}^{R}$: FRT with rank data based variance differences, FRT-PI: Plug in FRT with KS statistic, FRT-CI: repeated Plug in with KS-statistic, $T_{\textrm{sub}}$: Subsampling based quantile test

We used the functions of Ding et al. (2016) to compute the FRT-PI and the FRT-CI, respectively. For both tests, we proceeded the same way as for $T_{V}$, but with the difference that we followed the suggestions of Ding et al. (2016) and set the number of randomization samples to *B* = 500. In case of FRT-CI, a grid of 150 values was used to compute the maximum *p* value (this is the default value in the functions of Ding et al. 2016). $T_{\gamma }$ was obtained by regressing the treatment variable on the outcome variable in a regression model. Furthermore, the residual variance of this model (see Eq. 10) was also modeled as function of the treatment variable and the latter coefficient was used to test for heterogeneous treatment effects. Specifically, if the absolute value of the estimate divided by its standard error was greater than 1.96, the null hypothesis of a constant treatment effect was counted as rejected. The heterogeneous regression model was estimated using remlscore function in the R package statmod (Giner & Smyth, 2016). We used Mplus (Muthén & Muthén, 1998-2017) to compute the random coefficient regression model to obtain $T_{\beta }$. Mplus implements a maximum likelihood estimator for the model’s parameters (see, Muthén et al., 2017. In the simulation, we used the R package MplusAutomation (Hallquist & Wiley, 2018) to control Mplus from R. To obtain $T_{LR}$, we fitted two multiple group structural equation models with the lavaan package (Rosseel, 2012). The first model was an unconstrained model, in which the variance of the outcome variable in the experimental and the control group were allowed to differ, and in the second, constrained model, they were restricted to be equal. The LR-test is then obtained by comparing the fit of the two models with a chi-square difference test. Finally, we used the functions of Chernozhukov and Fernandez-Val (2005) with their default settings to obtain $T_{\textrm{sub}}$ (resulting in a subsampling size of $b \approx$ 20).

In each replication, we determined whether a test statistic rejected the null hypothesis of a constant treatment effect and used this information to calculate the rejection rate per simulation condition. When the standard deviation $\sigma _{\tau }$ is 0, this rate measures the type 1 error rate. If $\sigma _{\tau }$ is greater than 0, the rejection rate is an indicator of the power of a test.

**Table 3:** Rejection rates for different heterogeneous treatment effect tests depending on the distribution of the potential outcomes, amount of treatment effect variation ($\sigma _{\tau }$), and sample size when the average treatment effect is zero and covariates are considered

		$\sigma _{\tau }$ = 0				$\sigma _{\tau }$ = 0.25				$\sigma _{\tau }$ = 0.50			
Distribution	Test	25	50	125	250	25	50	125	250	25	50	125	250
*N*(0,1)	$T_{\gamma }$	0.048	0.050	0.048	0.034	0.197	0.340	0.585	0.937	0.485	0.791	0.987	1.000
	$T_{V}$	0.041	0.043	0.048	0.035	0.172	0.316	0.557	0.939	0.424	0.757	0.985	1.000
	$T_{V}^{R}$	0.050	0.048	0.054	0.038	0.153	0.253	0.453	0.862	0.355	0.646	0.929	1.000
	$T_{LR}$	0.054	0.052	0.053	0.034	0.213	0.347	0.590	0.938	0.504	0.797	0.987	1.000
	$T_{\beta }$	0.030	0.026	0.021	0.016	0.190	0.329	0.576	0.943	0.483	0.774	0.988	1.000
	$T_{D}$	0.043	0.043	0.048	0.035	0.173	0.320	0.557	0.939	0.426	0.757	0.985	1.000
	$T_{D}^{R}$	0.050	0.047	0.054	0.038	0.150	0.256	0.453	0.863	0.350	0.650	0.929	1.000
	FRT-PI	0.051	0.045	0.037	0.047	0.088	0.133	0.212	0.591	0.176	0.367	0.639	0.983
	FRT-CI	0.012	0.023	0.029	0.036	0.023	0.078	0.166	0.523	0.061	0.280	0.577	0.974
	$T_{\textrm{sub}}$	0.002	0.007	0.018	0.027	0.013	0.055	0.168	0.566	0.048	0.259	0.661	0.991
*t*(5)	$T_{\gamma }$	0.190	0.189	0.213	0.256	0.304	0.389	0.562	0.825	0.496	0.710	0.911	0.992
	$T_{V}$	0.067	0.043	0.046	0.064	0.122	0.189	0.324	0.619	0.307	0.519	0.784	0.962
	$T_{V}^{R}$	0.059	0.053	0.061	0.063	0.114	0.197	0.365	0.773	0.309	0.554	0.860	0.998
	$T_{LR}$	0.202	0.199	0.216	0.257	0.317	0.393	0.564	0.826	0.508	0.716	0.914	0.992
	$T_{\beta }$	0.034	0.025	0.022	0.031	0.134	0.178	0.299	0.599	0.297	0.492	0.741	0.946
	$T_{D}$	0.068	0.043	0.048	0.064	0.126	0.191	0.322	0.620	0.306	0.517	0.784	0.962
	$T_{D}^{R}$	0.062	0.050	0.061	0.063	0.113	0.198	0.366	0.773	0.313	0.559	0.859	0.998
	FRT-PI	0.044	0.043	0.040	0.039	0.088	0.111	0.148	0.353	0.143	0.233	0.429	0.899
	FRT-CI	0.009	0.021	0.020	0.025	0.027	0.055	0.112	0.314	0.045	0.153	0.368	0.865
	$T_{\textrm{sub}}$	0.006	0.007	0.007	0.010	0.013	0.024	0.073	0.228	0.039	0.139	0.331	0.828
log-*N*	$T_{\gamma }$	0.500	0.554	0.619	0.647	0.542	0.601	0.684	0.745	0.608	0.677	0.763	0.871
	$T_{V}$	0.053	0.059	0.058	0.057	0.083	0.109	0.114	0.156	0.173	0.197	0.284	0.419
	$T_{V}^{R}$	0.050	0.052	0.061	0.043	0.269	0.523	0.874	0.999	0.595	0.906	0.998	1.000
	$T_{LR}$	0.507	0.558	0.620	0.650	0.552	0.603	0.686	0.745	0.616	0.678	0.764	0.872
	$T_{\beta }$	0.027	0.028	0.030	0.025	0.060	0.080	0.070	0.097	0.104	0.130	0.189	0.320
	$T_{D}$	0.049	0.057	0.055	0.057	0.102	0.117	0.119	0.160	0.213	0.230	0.303	0.423
	$T_{D}^{R}$	0.054	0.053	0.061	0.042	0.265	0.514	0.869	0.999	0.577	0.901	0.997	1.000
	FRT-PI	0.164	0.119	0.081	0.079	0.198	0.230	0.261	0.426	0.310	0.387	0.567	0.860
	FRT-CI	0.029	0.032	0.034	0.037	0.048	0.105	0.152	0.333	0.080	0.199	0.399	0.791
	$T_{\textrm{sub}}$	0.004	0.014	0.011	0.008	0.010	0.023	0.025	0.055	0.014	0.042	0.095	0.377

Note. *N*(0,1) = standard normal distribution, *t*(5) = *t*-distribution with 5 degrees of freedom, log-*N* = log-normal distribution. $T_{\gamma }$: variance ratio test with heterogeneous regression model, $T_{V}$: FRT with raw data based variance ratios, $T_{V}^{R}$: FRT with rank data based variance ratios, $T_{LR}$: variance difference test with LR-test from multiple group SEM, $T_{\beta }$: variance difference test with random coefficient regression model, $T_{D}$: FRT with raw data based variance differences, $T_{D}^{R}$: FRT with rank data based variance differences, FRT-PI: Plug in FRT with KS statistic, FRT-CI: repeated Plug in with KS-statistic, $T_{\textrm{sub}}$: Subsampling based quantile test

## Results

We first discuss the results for the case that no covariate was considered in the tests. The results for the different tests concerning the two error rates were very similar for an average treatment effect of zero and one. Therefore, we focus on the results for the conditions of a zero average treatment effect here and later discuss some noticeable differences from the conditions in which the treatment effect is one.

Table 2 presents the results concerning type 1 error and power rates when no covariate was considered when performing the tests. Turning to the number of type 1 errors first (i.e., the columns with $\sigma _{\tau } = 0$), $T_{V}$ $T_{V}^{R}$, $T_{D}$, $T_{D}^{R}$, $T_{\gamma }$, $T_{LR}$ and the FRT-PI yielded exact or almost exact rejection rates when the potential outcomes were normally distributed, irrespective of the size of the two groups. For FRT-CI, $T_{\textrm{sub}}$, and $T_{\beta }$ the rates were too conservative, but they almost approached the nominal $\alpha$-level as the sample size increased. When the data followed a *t*-distribution, all FRT-based except FRT-CI tests (i.e., $T_{V}$, $T_{V}^{R}$, $T_{D}$, $T_{D}^{R}$, FRT-PI) were still near the nominal value. For the latter, the type 1 error rate again approached the nominal value with an increasing sample size, while the rates for $T_{\textrm{sub}}$ and $T_{\beta }$ remained largely too conservative. For $T_{\gamma }$ and $T_{LR}$, rejection rates were too liberal. In case of the log-normal distribution, $T_{\textrm{sub}}$ and $T_{\beta }$ yielded even higher type 1 error rates. Interestingly, while FRT-PI showed a good size for normally and *t*-distributed potential outcomes, the test was too liberal when the potential outcomes were log-normally distributed and when the samples size was small to moderate. In this case, the FRT-CI yielded more exact rejection rates. Finally, $T_{V}$ $T_{V}^{R}$, $T_{D}$, and $T_{D}^{R}$ were again near the nominal value, and the rates for $T_{\textrm{sub}}$ and $T_{\beta }$ were again largely unaffected by the sample size.

With regard to the power of the tests (see columns with $\sigma _{\tau } = 0.25$ and $\sigma _{\tau } = 0.50$ in Table 2), all tests were more powerful the larger the size of the effect and the larger the size of the two groups. However, there were notable differences between the tests depending on conditions. Specifically, $T_{V}$, $T_{D}$, $T_{\gamma }$, $T_{LR}$, and $T_{\beta }$ were the most powerful tests in case of normally distributed data, irrespective of sample size and level of treatment effect variation. The rank-based FRT versions $T_{V}^{R}$ and $T_{D}^{R}$ were always less powerful than their raw-data counterparts $T_{D}$ and $T_{V}$, respectively, and $T_{\textrm{sub}}$ was more powerful than FRT-CI, but not FRT-PI. However, the latter three tests reached power rates above 80% only at 250 participants per group and when treatment effect variation was large. All other tests reached these values prior to these three tests when treatment effect variation was moderate and large, but even these tests did not reach a sufficient power for the small sample size condition. A similar result pattern occurred in conditions in which the potential outcomes were *t*-distributed. Again, $T_{V}$, $T_{D}$, $T_{\gamma }$, $T_{LR}$ and $T_{\beta }$ were the most powerful tests, although the result for $T_{\gamma }$ and $T_{LR}$ has to be considered in light of the large type 1 error rates when there was no effect variation. Interestingly, $T_{\beta }$, although also based on normal-theory, performed similar to $T_{V}$ and $T_{D}$. Furthermore, and in contrast to the normal distribution condition, FRT-PI was more powerful than FRT-CI and $T_{\textrm{sub}}$ and the rank-based versions of $T_{V}$ and $T_{D}$ had the same power rates as their raw-data counterparts. Finally, when the potential outcomes were log-normally distributed, $T^{R}_{V}$, $T^{R}_{D}$, $T_{\gamma }$, $T_{LR}$ and FRT-PI were the most powerful tests. Again, for $T_{\gamma }$ and $T_{LR}$ this result has to be considered in light of the worse performance of the two tests in case of no treatment effect variation. Similarly, the results concerning the rank-based tests should be interpreted in comparison to their performance when the average treatment effect is one, where $T^{R}_{V}$ and $T^{R}_{D}$ yielded high rejection rates when the treatment effect was constant (see the discussion below).

The type 1 and type 2 error rates of the tests when considering an additional covariate were, unexpectedly, very similar to the error rates when not considering the covariate (see Table 3 for the specific results). When we examine the small sample size condition only (i.e., 25 participants per condition) and average across all tests, we find that for normally distributed data the mean rejection rate is 0.038 when $\sigma _{\tau }$ is zero, it is .137 when $\sigma _{\tau }$ is 0.25, and it is 0.331 when $\sigma _{\tau }$ is 0.50. These values differ only slightly from the values that we obtain when we consider the tests without covariates, $\sigma _{\tau }$ = 0: 0.038, $\sigma _{\tau }$ = 0.25: .133, $\sigma _{\tau }$ = 0.50: 0.333. Very similar results are obtained when we consider the conditions in which the data was *t*- or log-normal-distributed, and also when we consider each specific tests. Thus, in the conditions studied here, taking a covariate into account leads, if at all, only to a small improvement in the power of the tests, when the correlation between the covariate and the dependent variable does not exceed 0.3.

**Table 4:** Rejection Rates for a raw-data ($T_{Raw}$) and a rank-data based FRT for the average treatment effect depending on the amount of treatment effect variation ($\sigma _{\tau }$) and sample size (*n*) when the data are log-normally distributed

		$\tau$ = 0		$\tau$ = 1	
$\sigma _{\tau }$	*n*	$T_{Raw}$	$T_{Rank}$	$T_{Raw}$	$T_{Rank}$
0.00	50	0.050	0.037	0.517	0.898
	100	0.059	0.067	0.742	0.998
	200	0.040	0.028	0.912	1.000
	500	0.055	0.056	0.997	1.000
0.25	50	0.052	0.108	0.417	0.770
	100	0.049	0.190	0.632	0.969
	200	0.058	0.328	0.835	1.000
	500	0.054	0.688	0.989	1.000
0.50	50	0.051	0.216	0.354	0.515
	100	0.041	0.369	0.559	0.776
	200	0.045	0.659	0.811	0.953
	500	0.050	0.973	0.981	1.000

Finally, as mentioned above, the results are very similar for the conditions in which the average treatment effect is one (see Tables 6 and 7 in the Appendix for the specific error rates of the considered tests). Exceptions were the results for the rank tests $T^{R}_{V}$ and $T^{R}_{D}$ when the data were log-normally distributed. Here, rejection rates were unacceptably high when there was *no* treatment effect variation (i.e., $\sigma _{\tau }$ = 0). To better understand these results, we conducted another small simulation study in which we examined the performance of a FRT when testing the null hypotheses that the *average treatment effect* is zero for log-normal data. The results showed (see Table 4) that the rank based test performed better than the raw-data based test when there was no treatment effect heterogeneity (i.e., $\sigma _{\tau }$ = 0). However, when the treatment effect was zero, we found that rejection rates of the rank test increased the larger the variation in the treatment effect, while the raw-data based test had the correct size. This suggests that nonzero treatment effect heterogeneity results in a difference between the two outcome means in the rank data, which is actually not present, and this increases with higher heterogeneity. This ’bias’ also affects the rank-test of the variances (analogous to the test using the coefficient of variation, as discussed above). Finally, when the average treatment is one, then the actual difference in means is ’added’ to this bias, leading to the exceptional rejection rates.

## Discussion

Psychological researchers are increasingly interested in methods that allow them to detect whether a treatment effect investigated in a study is non-constant across participants. Based on the potential outcome framework, a number of procedures were suggested to test the null hypothesis of a homogeneous treatment effect. These tests can be distinguished according to whether they make distributional assumptions and to which sampling statistics are used in the test. The present study was done to examine the type 1 and type 2 error rates of the suggested tests as a function of the amount of treatment heterogeneity, the presence of an average causal effect, the distribution of the potential outcomes, the sample size, and of whether a covariate is considered when conducting the test.

With regard to the type 1 error rate, our results replicate the findings of earlier simulation research (Bloome & Schrage, 2021; Ding et al., 2016) by showing that the majority of tests were close to the nominal $\alpha$-level regardless of sample size when the data was normally distributed. However, extending prior research we also found that the variance tests in the heterogeneous regression model $T_{\gamma }$ and the LR test implemented with a multiple group structural equation model $T_{LR}$ rejected the null hypothesis too often in the case of non-normally distributed data. Furthermore, we found that the subsampling procedure $T_{sub}$ and the test in the random coefficient regression model $T_{\beta }$ decided too conservatively in these conditions, and that the FRT-CI performed best in case of skewed data. Overall, these results suggest that, regardless of sample size, applied researchers should use a Fisher randomization test with variance ratios or variance differences (i.e., $T_{V}$, $T_{D}$) or the empirical distribution function (i.e., FRT-CI), because they protect well against false positive decisions. When the data are normally distributed, they could, again regardless of sample size, use the variance tests in the heterogeneous regression model $T_{\gamma }$ and the LR test, but should use one of the FRT tests as a sensitivity check if there is any doubt as to whether the normality assumption is met.

Concerning the power of the tests, the results of the simulation – at least for the small sample sizes of 25 and 50 persons per group – are quite sobering, because none of the tests reached satisfactory power levels even when size of treatment heterogeneity was stronger. When samples sizes were larger, our results were consistent with earlier research showing that the variance test of the heterogeneous regression model is characterized by good type 2 error rates in case of normally distributed data (Bloome & Schrage, 2021). They also show that the LR-test and the test of the random coefficient regression model achieved good power. Power rates of all three tests were slightly larger than the power rates of the variance ratio and the variance difference tests, but this is to be expected given that the parametric assumptions are met in these conditions. Furthermore, the power of FRT-PI increased with larger sample sizes and with larger treatment effect heterogeneity. The same pattern occurred for FRT-CI, although it performed better (in terms of type 2 *and* type 1 error rates) than FRT-PI in case of skewed data. For practitioners these results suggest that they should use the FRT variance tests, the variance test in the heterogeneous regression model $T_{\gamma }$ or the LR test when the data is normally distributed. However, they should keep in mind that the power is only acceptable for sample sizes greater than or equal to 50 participants per group in case of large heterogeneity in treatment effects, or with 250 participants per group in case of moderate heterogeneity. In the case of non-normally distributed data, they should use the FRT variance tests or the FRT-PI test. Once again, however, when interpreting the result, it is important to bear in mind that the power is only sufficiently high for very large samples (250 people per group in case of high heterogeneity).

We also examined the performance of rank-based versions of the variance ratio and the variance difference tests, because in the case of the average treatment effect, simulation studies show that an FRT that uses rank data (i.e., the initial data is transformed into ranks in a first step) performs well across many simulation conditions (Imbens & Rubin, 2015; Rosenbaum, 2002, 2010). In our simulations, these tests performed similar (in case of *t*-distributed data) or worse (in case of normally distributed data) than their raw-data based counterparts. Furthermore, in the case of log-normal distributed data, their performance was unsatisfactory. Thus, we provisionally conclude that the performance advantage that is observed for the average treatment effect does not generalize to the detection of a heterogeneous treatment effect. However, our results have to be replicated in future simulation studies to reach a final conclusion.

Finally, we also investigated whether the power of the tests is increased by considering a relevant covariate. However, our results show only a tiny effect of including a covariate. The obvious explanation is that the influence of the covariate was too small in our simulation to detect an effect on power. We used a moderate effect, because we think this is the most plausible value for the considered setting in which participants are randomly assigned to treatment. Nevertheless, future research should replicate and also extend our results by additionally examining larger sizes of the covariate’s effect. Another point to consider is that covariates are often not measured with perfect reliability, which in turn may also affect the error rates of the tests for treatment effect heterogeneity. In fact, we assumed here that the outcome variable is not subject to measurement error and it remains unclear how a violation of this assumption would affect the different tests. We think that this is also an interesting question to examine in future research.

Furthermore, in our simulation study, we focused on tests that have been proposed in the causal inference literature to test for treatment effect heterogeneity. However, in the context of methods that compare variance terms, the statistical literature suggests a number of further tests that are aimed to be more robust to violations of the normal distribution, such as the Levene test or the Brown and Forsythe test (see Wilcox, 2017b, for an overview) or tests that are based on bootstrapping (see Lim & Loh, 1996; Wilcox, 2017a). We expect these tests to perform similarly to the FRT tests, though this assumption should be validated in a future simulation study. Finally, in the case of the average treatment effect, previous research has found that the meta-analytical integration of the results of many small-sample studies can match a single, large-sample study in terms of power (e.g., Cappelleri et al., 1996). This suggests that the pooled results of several heterogeneous treatment effect tests may have a sufficient power, even when the single studies are underpowered due to using small samples. We think that testing this hypothesis in future research is not only interesting from an applied perspective, but also from a methodological point of view: Although meta-analytic methods for the variance ratio statistic exist (see Nakagawa et al., 2015; Senior et al., 2020)), it is currently unclear, at least to our knowledge, how to best pool the results of the FRT tests or the quantile tests (i.e., whether Fisher’s or Stouffer’s method is appropriate, for example, when there is substantial between-study heterogeneity).^4^

To summarize, our results suggest that researchers often fail to reject the null hypothesis of a constant treatment effect in a single study even when there is actual heterogeneity in effects present in the data. We think that this result is relevant for applied researchers from multiple points of view. To begin with, currently the sample sizes are determined a priori in such a way that a pre-specified average (causal) effect can be tested with as much power as possible. Thus, assuming that future studies will extend their focus to treatment effect heterogeneity, much larger samples sizes have to be assessed, at least when the variables that are responsible for treatment effect heterogeneity are not known before conducting the RCT.

**Table 5:** Rejection rates for a variance ratio test ($T_{R}$), a variance difference test ($T_{D}$), and an interaction test ($T_{I}$) depending on the size of the sample (*n*)

*n*	$T_{R}$	$T_{D}$	$T_{I}$
25	0.077	0.077	0.375
50	0.121	0.125	0.670
100	0.176	0.178	0.928
250	0.435	0.434	1.000

Specifically, in our study we examined the setting that researchers have not assessed or do not know the heterogeneity-generating variables and therefore perform one of the (global) tests considered here. However, researchers may sometimes know these variables prior to the randomization and may then, assuming that they have assessed them and that a linear model is true, compute a regression model with an interaction term to test whether the treatment effect varies with this variable. In this case, the power of the interaction test is larger than the power of the tests considered in the simulation. For instance, when the population model is linear and the interaction term has a moderate effect ($R^2$ about .13), the power of the interaction test is higher than the power of the variance ratio and the variance difference tests (see Table 5).^5^ For applied researchers this result suggests, second, that they should already consider which variables can explain potential variation in the average treatment effect during the planning stage of the study, because in this case a much higher power can be achieved at lower samples sizes.

**Table 6:** [XMLCONT]

		$\sigma _{\tau }$ = 0				$\sigma _{\tau }$ = 0.25				$\sigma _{\tau }$ = 0.50			
Distribution	Test	25	50	125	250	25	50	125	250	25	50	125	250
	FRT-PI	0.206	0.134	0.102	0.066	0.238	0.236	0.312	0.432	0.340	0.402	0.614	0.846
	FRT-CI	0.018	0.040	0.050	0.028	0.032	0.080	0.176	0.292	0.040	0.146	0.472	0.768
	$T_{\textrm{sub}}$	0.010	0.011	0.009	0.010	0.015	0.030	0.029	0.067	0.024	0.055	0.106	0.396

Note. *N*(0,1) = standard normal distribution, *t*(5) = *t*-distribution with 5 degrees of freedom, log-*N* = log-normal distribution. $T_{\gamma }$: variance ratio test with heterogeneous regression model, $T_{V}$: FRT with raw data based variance ratios, $T_{V}^{R}$: FRT with rank data based variance ratios, $T_{LR}$: variance difference test with LR-Test from multiple group SEM, $T_{\beta }$: variance difference test with random coefficient regression model, $T_{D}$: FRT with raw data based variance differences, $T_{D}^{R}$: FRT with rank data based variance differences, FRT-PI: Plug in FRT with KS statistic, FRT-CI: repeated Plug in with KS-statistic, $T_{\textrm{sub}}$: Subsampling based quantile test

A final recommendation for applied researchers pertains to the case that one of the tests studied here leads to the rejection of the null hypothesis of a constant treatment effect. Then, researchers will be motivated to detect the person-level variables that explain differences in the treatment effect. In the introduction we mentioned that there are a number of statistical approaches available (classical as well as more modern approaches from machine learning) that can then be used for this task. When performing these (post-hoc) analyses, however, it has to be taken into account that one potentially conducts a large number of statistical tests, which may lead to many false-positive results (Schulz and Grimes, 2005; Sun et al., 2014). Thus, one has to investigate in other studies how stable (in terms of replicability) and generalizable (in terms of external validity) the resulting findings are, also given that the sample sizes are (typically) optimized with respect to the statistical test of the average treatment effect.

To conclude, we conducted a simulation study to investigate the type 1 and type 2 error rates of different tests of treatment effect heterogeneity that were suggested in the causal inference literature. The results suggest that a randomization test should be used in order to have good control of the type 1 error. Furthermore, all tests studied are associated with high type 2 error rates when sample sizes are small to moderate. Thus, to detect heterogeneous treatment effects with sufficient power, much larger samples are needed than those typically collected in current studies, or new test procedures must be developed that have higher power even with smaller samples.

## Appendix

### Additional results

Here, we present additional results of the simulation study. Table 6 displays the results for the examined tests when no cova-riates are considered and when the average treatment effect is 1, and Table 7 shows the results for the tests with covariates considered and for an average treatment effect of 1.

**Table 7:** Rejection rates for different heterogeneous treatment effect tests depending on the distribution of the potential outcomes, amount of treatment effect variation ($\sigma _{\tau }$), and sample size when the average treatment effect is one and no covariates are considered

		$\sigma _{\tau }$ = 0				$\sigma _{\tau }$ = 0.25				$\sigma _{\tau }$ = 0.50			
Distribution	Test	25	50	125	250	25	50	125	250	25	50	125	250
*N*(0,1)	$T_{\gamma }$	0.064	0.046	0.038	0.048	0.198	0.331	0.612	0.933	0.486	0.818	0.978	1.000
	$T_{V}$	0.057	0.048	0.046	0.051	0.183	0.319	0.600	0.934	0.447	0.802	0.979	1.000
	$T_{V}^{R}$	0.045	0.043	0.039	0.043	0.128	0.235	0.416	0.787	0.319	0.617	0.894	0.999
	$T_{LR}$	0.068	0.049	0.039	0.048	0.202	0.336	0.613	0.933	0.490	0.820	0.978	1.000
	$T_{\beta }$	0.035	0.026	0.022	0.026	0.195	0.318	0.588	0.930	0.466	0.799	0.980	1.000
	$T_{D}$	0.020	0.016	0.012	0.007	0.096	0.198	0.430	0.866	0.325	0.683	0.947	1.000
	$T_{D}^{R}$	0.017	0.013	0.015	0.015	0.072	0.147	0.279	0.644	0.224	0.477	0.827	0.998
	FRT-PI	0.041	0.048	0.055	0.050	0.072	0.130	0.262	0.523	0.162	0.311	0.736	0.980
	FRT-CI	0.006	0.016	0.025	0.033	0.015	0.067	0.195	0.456	0.044	0.206	0.652	0.970
	$T_{\textrm{sub}}$	0.010	0.019	0.039	0.032	0.029	0.118	0.307	0.679	0.106	0.394	0.750	0.996
*t*(5)	$T_{\gamma }$	0.186	0.191	0.222	0.227	0.308	0.377	0.560	0.814	0.520	0.727	0.896	0.991
	$T_{V}$	0.082	0.075	0.073	0.068	0.157	0.228	0.359	0.638	0.357	0.567	0.790	0.964
	$T_{V}^{R}$	0.068	0.059	0.063	0.074	0.133	0.195	0.380	0.697	0.298	0.536	0.815	0.996
	$T_{LR}$	0.191	0.194	0.222	0.227	0.315	0.381	0.560	0.814	0.523	0.729	0.897	0.991
	$T_{\beta }$	0.024	0.028	0.022	0.019	0.113	0.169	0.282	0.586	0.285	0.483	0.713	0.945
	$T_{D}$	0.036	0.028	0.026	0.030	0.104	0.162	0.258	0.558	0.272	0.483	0.734	0.956
	$T_{D}^{R}$	0.032	0.030	0.021	0.029	0.093	0.132	0.280	0.612	0.228	0.436	0.739	0.993
	FRT-PI	0.064	0.040	0.046	0.051	0.075	0.096	0.179	0.368	0.107	0.230	0.542	0.887
	FRT-CI	0.007	0.009	0.029	0.038	0.017	0.043	0.115	0.291	0.024	0.133	0.445	0.844
	$T_{\textrm{sub}}$	0.008	0.013	0.010	0.014	0.028	0.045	0.091	0.282	0.085	0.205	0.343	0.863
log-*N*	$T_{\gamma }$	0.538	0.555	0.624	0.652	0.560	0.595	0.651	0.739	0.568	0.690	0.760	0.854
	$T_{V}$	0.129	0.074	0.085	0.065	0.111	0.092	0.118	0.171	0.121	0.183	0.269	0.428
	$T_{V}^{R}$	0.823	0.992	1.000	1.000	0.496	0.829	0.989	1.000	0.111	0.186	0.322	0.619
	$T_{LR}$	0.539	0.555	0.625	0.652	0.562	0.596	0.651	0.739	0.573	0.692	0.762	0.854
	$T_{\beta }$	0.026	0.021	0.027	0.021	0.071	0.050	0.070	0.101	0.088	0.116	0.173	0.303
	$T_{D}$	0.106	0.055	0.052	0.050	0.041	0.048	0.075	0.127	0.041	0.128	0.223	0.386
	$T_{D}^{R}$	0.604	0.940	1.000	1.000	0.348	0.691	0.965	1.000	0.075	0.139	0.256	0.558
	FRT-PI	0.206	0.134	0.102	0.066	0.238	0.236	0.312	0.432	0.340	0.402	0.614	0.846
	FRT-CI	0.018	0.040	0.050	0.028	0.032	0.080	0.176	0.292	0.040	0.146	0.472	0.768
	$T_{\textrm{sub}}$	0.010	0.011	0.009	0.010	0.015	0.030	0.029	0.067	0.024	0.055	0.106	0.396

Note. *N*(0,1) = standard normal distribution, *t*(5) = *t*-distribution with 5 degrees of freedom, log-*N* = log-normal distribution. $T_{\gamma }$: variance ratio test with heterogeneous regression model, $T_{V}$: FRT with raw data based variance ratios, $T_{V}^{R}$: FRT with rank data based variance ratios, $T_{LR}$: variance difference test with LR-Test from multiple group SEM, $T_{\beta }$: variance difference test with random coefficient regression model, $T_{D}$: FRT with raw data based variance differences, $T_{D}^{R}$: FRT with rank data based variance differences, FRT-PI: Plug in FRT with KS statistic, FRT-CI: repeated Plug in with KS-statistic, $T_{\textrm{sub}}$: Subsampling based quantile test

**Table 8:** Rejection rates for different heterogeneous treatment effect tests depending on the distribution of the potential outcomes, amount of treatment effect variation ($\sigma _{\tau }$), and sample size when the average treatment effect is one and with covariates considered

		$\sigma _{\tau }$ = 0				$\sigma _{\tau }$ = 0.25				$\sigma _{\tau }$ = 0.50			
Distribution	Test	25	50	125	250	25	50	125	250	25	50	125	250
*N*(0,1)	$T_{\gamma }$	0.062	0.067	0.057	0.037	0.208	0.328	0.600	0.937	0.511	0.783	0.983	1.000
	$T_{V}$	0.063	0.068	0.059	0.044	0.195	0.323	0.587	0.933	0.453	0.762	0.976	1.000
	$T_{V}^{R}$	0.047	0.057	0.038	0.043	0.142	0.194	0.439	0.778	0.312	0.553	0.882	0.998
	$T_{LR}$	0.075	0.070	0.061	0.038	0.226	0.334	0.605	0.938	0.532	0.788	0.982	1.000
	$T_{\beta }$	0.033	0.031	0.036	0.019	0.205	0.334	0.586	0.933	0.487	0.773	0.975	1.000
	$T_{D}$	0.022	0.024	0.026	0.012	0.094	0.189	0.432	0.874	0.320	0.628	0.940	1.000
	$T_{D}^{R}$	0.017	0.014	0.011	0.017	0.083	0.115	0.292	0.649	0.224	0.439	0.798	0.995
	FRT-PI	0.045	0.033	0.041	0.042	0.085	0.139	0.212	0.561	0.192	0.325	0.665	0.986
	FRT-CI	0.009	0.013	0.025	0.026	0.032	0.094	0.167	0.503	0.075	0.240	0.604	0.976
	$T_{\textrm{sub}}$	0.003	0.008	0.020	0.020	0.007	0.049	0.215	0.588	0.040	0.254	0.675	0.991
*t*(5)	$T_{\gamma }$	0.179	0.169	0.219	0.233	0.278	0.404	0.581	0.830	0.491	0.740	0.882	0.992
	$T_{V}$	0.076	0.074	0.072	0.073	0.145	0.241	0.406	0.665	0.339	0.570	0.783	0.970
	$T_{V}^{R}$	0.057	0.064	0.066	0.079	0.124	0.202	0.399	0.691	0.283	0.536	0.806	0.991
	$T_{LR}$	0.187	0.177	0.221	0.233	0.292	0.412	0.585	0.830	0.510	0.744	0.882	0.992
	$T_{\beta }$	0.041	0.023	0.017	0.020	0.119	0.185	0.342	0.605	0.294	0.506	0.730	0.959
	$T_{D}$	0.037	0.031	0.035	0.040	0.097	0.157	0.310	0.579	0.251	0.485	0.730	0.957
	$T_{D}^{R}$	0.027	0.031	0.025	0.032	0.081	0.131	0.305	0.605	0.216	0.445	0.740	0.985
	FRT-PI	0.045	0.044	0.047	0.055	0.086	0.090	0.162	0.386	0.146	0.247	0.431	0.892
	FRT-CI	0.008	0.019	0.032	0.040	0.024	0.048	0.116	0.328	0.055	0.153	0.366	0.856
	$T_{\textrm{sub}}$	0.003	0.008	0.008	0.017	0.013	0.037	0.105	0.248	0.032	0.153	0.348	0.853
log-*N*	$T_{\gamma }$	0.533	0.575	0.600	0.668	0.534	0.606	0.687	0.769	0.586	0.666	0.766	0.883
	$T_{V}$	0.086	0.079	0.091	0.076	0.086	0.092	0.133	0.176	0.129	0.178	0.269	0.461
	$T_{V}^{R}$	0.688	0.965	1.000	1.000	0.337	0.667	0.947	1.000	0.089	0.113	0.233	0.482
	$T_{LR}$	0.550	0.580	0.602	0.668	0.547	0.611	0.689	0.769	0.597	0.670	0.769	0.883
	$T_{\beta }$	0.035	0.020	0.028	0.022	0.057	0.055	0.084	0.113	0.102	0.120	0.176	0.335
	$T_{D}$	0.072	0.052	0.058	0.049	0.037	0.037	0.083	0.136	0.063	0.128	0.235	0.427
	$T_{D}^{R}$	0.486	0.892	0.995	1.000	0.233	0.546	0.901	1.000	0.061	0.090	0.177	0.419
	FRT-PI	0.168	0.123	0.117	0.096	0.207	0.218	0.290	0.409	0.351	0.367	0.557	0.841
	FRT-CI	0.027	0.035	0.049	0.049	0.050	0.072	0.179	0.325	0.098	0.187	0.408	0.776
	$T_{\textrm{sub}}$	0.003	0.008	0.009	0.007	0.009	0.017	0.024	0.072	0.017	0.042	0.111	0.412

Note. *N*(0,1) = standard normal distribution, *t*(5) = *t*-distribution with 5 degrees of freedom, log-*N* = log-normal distribution. $T_{\gamma }$: variance ratio test with heterogeneous regression model, $T_{V}$: FRT with raw data based variance ratios, $T_{V}^{R}$: FRT with rank data based variance ratios, $T_{LR}$: variance difference test with LR-test from multiple group SEM, $T_{\beta }$: variance difference test with random coefficient regression model, $T_{D}$: FRT with raw data based variance differences, $T_{D}^{R}$: FRT with rank data based variance differences, FRT-PI: Plug in FRT with KS statistic, FRT-CI: repeated Plug in with KS-statistic, $T_{\textrm{sub}}$: Subsampling based quantile test

## Footnotes

1 We also computed $T_{CV}$ in selected conditions of our simulation. When the average treatment effect and the variance of the treatment effects was 0, the rejection rate of $T_{CV}$ was near the nominal value (i.e., 0.05). However, when the average treatment effect was 1, but the variance of the treatment effects was 0, the rejection rate was 0.935.

2 The heterogeneous regression model, the random coefficient model, and the structural equation model assume a linear relationship between the covariates and the outcome. When this assumption is suspected to be violated, one could also first predict the outcome with the covariates using any machine learning model and then proceed with the residuals from this model.

3 Chernozhukov and Fernandez (2005) also suggest a bootstrap-variant of their approach that we also included in the simulation. However, the performance of the test with regard to the type 1 and type 2 error rates was unsatisfactory (see Ding et al., 2016, for similar findings), so that we decided to not report results concerning this test.

4 In a preliminary simulation study, we examined the power of a pooled variance ratio test depending on the size of treatment effect heterogeneity in the single studies, the number of the studies to be pooled, and the amount of between-study heterogeneity. Specifically, to generate the data for a single study, we used the same population model as in the simulation study reported in the main text. The differences were that we generated data for a small, a moderate, and a large heterogeneous treatment effect (i.e., the standard deviation of $\sigma _{\tau }$ was set to 0.10, 0.25, or 0.50; see Eq. 21), and that we considered the small sample size condition only (i.e., 25 persons in the experimental and the control group in a single study). In addition, we varied the amount of systematic variation of the heterogeneous treatment effect across studies (i.e., the between-study heterogeneity was either 0 or 0.10) and we varied the number of studies that are meta-analytically integrated (i.e., 10 vs. 30 vs. 50 studies). In each of the 1000 replications generated for a simulation condition, we computed a random-effect meta-analysis (using the metafor package, see Viechtbauer, 2010) and checked whether the pooled variance ratio was significantly different from zero. The results showed that when the size of the heterogeneous treatment effect was small, the power of the pooled test was adequate, when the number of aggregated studies was 50 and when there was no between-study heterogeneity (i.e., the power was .816). When between-study heterogeneity was 0.10, the power was lower than .80 when the number of to-be-aggregated studies was 50 (i.e., the power was .746). Furthermore, when the heterogeneous treatment effect was moderate or large, the power of the pooled variance ratio test was already adequate when the number of studies was 10 (i.e., the power was near 1.00 in almost all conditions), independent upon the amount of between-study heterogeneity. The exception was the moderate heterogeneous treatment effect condition in which between-study heterogeneity was 0.10; here, the nominal power level was not reached (i.e., the power was .750). However, we note that these results were obtained under rather optimal conditions (i.e., correct test statistic; distributional assumptions are met) and that they have to be replicated in future simulation research.

5 The model used to generate the data was

23 $$\begin{aligned} Y = 0.5 \cdot A \cdot X + \epsilon \end{aligned}$$

were *A* is the treatment variable (with prevalence 0.5) and *X* is a standard normal variable. The residual terms were also standard normally distributed.
